# The gene *transformer-2 *of *Sciara *(Diptera, Nematocera) and its effect on *Drosophila *sexual development

**DOI:** 10.1186/1471-213X-11-19

**Published:** 2011-03-15

**Authors:** Iker Martín, María F Ruiz, Lucas Sánchez

**Affiliations:** 1 Centro de Investigaciones Biológicas (C.S.I.C.), Ramiro de Maeztu 9, 28040 Madrid, Spain; 2 NEIKER-TEKNALIA, Berreaga 1, 48160-Derio, Vizcaya, Spain

**Keywords:** Sciara, sex determination, transformer-2 gene

## Abstract

**Background:**

The gene *transformer-2*, which is involved in sex determination, has been studied in *Drosophila, Musca*, *Ceratitis*, *Anastrepha *and *Lucilia*. All these members of Diptera belong to the suborder Brachycera. In this work, it is reported the isolation and characterisation of genes *transformer-2 *of the dipterans *Sciara ocellaris *and *Bradysia coprophila *(formerly *Sciara coprophila*), which belong to the much less extensively analysed *Sciaridae *Family of the Suborder Nematocera, which is paraphyletic with respect to Suborder Brachycera.

**Results:**

The *transformer-2 *genes of the studied *Sciara *species were found to be transcribed in both sexes during development and adult life, in both the soma and germ lines. They produced a single primary transcript, which follows the same alternative splicing in both sexes, giving rise to different mRNAs isoforms. In *S. ocellaris *the most abundant mRNA isoform encoded a full-length protein of 251 amino acids, while that of *B. coprophila *encoded a protein of 246 amino acids. Both showed the features of the SR protein family. The less significant mRNA isoforms of both species encoded truncated, presumably non-functional Transformer-2 proteins. The comparison of the functional *Sciara *Transformer-2 proteins among themselves and those of other insects revealed the greatest degree of conservation in the RRM domain and linker region. In contrast, the RS1 and RS2 domains showed extensive variation with respect to their number of amino acids and their arginine-serine (RS) dipeptide content. The expression of *S. ocellaris *Transformer-2 protein in *Drosophila *XX pseudomales lacking the endogenous *transformer-2 *function caused their partial feminisation.

**Conclusions:**

The *transformer-2 *genes of both *Sciaridae *species encode a single protein in both sexes that shares the characteristics of the Transformer-2 proteins of other insects. These proteins showed conserved sex-determination function in *Drosophila*; i.e., they were able to form a complex with the endogenous *Drosophila *Transformer protein that controls the female-specific splicing of the *Drosophila doublesex *pre-mRNA. However, it appears that the complex formed between the *Drosophila *Transformer protein and the *Sciara *Transformer-2 protein is less effective at inducing the female-specific splicing of the endogenous *Drosophila doublesex *pre-mRNA than the *Drosophila*Transformer-Transformer2 complex. This suggests the existence of species-specific co-evolution of the Transformer and Transformer-2 proteins.

## Background

Sex determination refers to the developmental programme that commits the embryo to following either the male or the female pathway. Recent years have seen a great amount of interest in the evolution of developmental mechanisms at the genetic and molecular levels, and in determining the evolutionary processes by which these mechanisms came into existence. Given the great variety of sex determination mechanisms, this process is exceptionally suitable for comparative study. Indeed, sex determination has long been of major interest not only as a developmental process but also because of the evolutionary problem it poses - a problem that can only be solved by identifying and comparing the genetic structures of sex determination pathways. Molecular genetic technologies now allow such comparisons to be made. In addition, sex determination in the *Drosophila melanogaster *reference system is known in fine detail, making truly informative comparisons possible.

The characterisation of the sex determination genes in *D. melanogaster *has shown that their control during development is governed by the sex-specific splicing of their products (reviewed in [1]). The product of a gene controls the sex-specific splicing of the pre-mRNA from the downstream gene in the genetic cascade. *Sex-lethal (Sxl) *is at the top of this cascade and acts as the memory device for female sexual development via its auto-regulatory function: its product controls the splicing of its own pre-mRNA [2,3]. In addition, Sxl controls the splicing of the pre-mRNA from the downstream gene *transformer (tra) *[4-6]. The Tra product and the product of the constitutive gene *transformer-2 (tra-2) *control the sex-specific splicing of pre-mRNA from the gene *doublesex (dsx) *[7-10], which is transcribed in both sexes but gives rise to two different proteins, DsxF and DsxM [11,12]. These are transcription factors that impose female and male sexual development respectively via the sex-specific regulation of the so-called sexual cytodifferentiation genes.

Genes homologous to the sex determination genes of *D. melanogaster *have been sought in other insects (reviewed in [13-15]). In the sciarid flies, which belong to the dipteran suborder Nematocera, only the orthologues of gene *Sxl *have been characterised in *Sciara ocellaris *[16], *Bradysia coprophila, Rynchosciara americana *and *Trichosia pubescens *[17]. The *Sxl *gene of these species is not regulated in a sex-specific fashion, and therefore the same *Sxl *transcript encoding the functional Sxl protein is found in both males and females. Thus, in the sciarids, *Sxl *does not appear to play the key discriminating role in sex determination that it plays in *Drosophila*.

Apart from in *D. melanogaster*, the gene *tra-2 *has been studied in *D. virilis *[18], and has been characterised in the housefly *Musca domestica *[19], in the tephritid *Ceratitis capitata *[20,21], in twelve *Anastrepha *species [22], and in the calliphorid *Lucilia cuprina *[23]. Outside the dipterans, *tra-2 *has been isolated in the lepidopteron *Bombyx mori *[24]. In all cases *tra-2 *is transcribed in both sexes during development, producing the same protein in males and females. The injection of *Musca tra-2 *dsRNA into *Musca *embryos results in the complete transformation of genotypically female embryos into fertile adult males, highlighting the role of *tra-2 *in *Musca *sex determination. This gene is required for the female-specific splicing of *Musca dsx *pre-mRNA. It also participates in the autocatalytic activity of gene *F *[19], i.e., the *Musca tra *orthologous gene [25], the key sex-determining gene in the housefly [26]. The injection of the respective *tra2-*dsRNA into *Ceratitis *[21] and *Anastrepha *[22] results in the destruction of endogenous *tra-2 *function in both species and the subsequent male-specific splicing of the endogenous *tra *and *dsx *pre-mRNAs, leading to the transformation of genotypically female embryos into adult pseudomales. This highlights the role of *tra-2 *in *Ceratitis *and *Anastrepha *sex determination.

The present paper reports the isolation and characterisation of *tra-2 *of *S. ocellaris *and *B. coprophila *(formerly *Sciara coprophila*). Their Tra2 proteins were compared to other known insect Tra2 proteins and the effect of *Sciara tra-2 *on *Drosophila *sex determination analysed.

## Results and discussion

### Molecular organisation of *tra-2 *in *S. ocellaris *and *B. coprophila*

The first step in the isolation of the *S. ocellaris tra-2 *gene (*Sotra2*) was to perform RT-PCR on total RNA from adults. Reverse transcription was performed using the primer oligo-dT, while two-nested PCR reactions were performed with three degenerated primers: Mar17, Mar26 and Tra2.B (the location and the sequences of the primers used in this work are shown in Additional file 1). The first PCR reaction was performed using the primer pair Mar26 plus Mar17, the second using Mar26 plus Tra2.B. An amplicon of 133 bp was amplified, cloned and sequenced. The conceptual amino acid sequence of this amplicon showed a high degree of similarity with the 3' region of the RRM domain of the *D. melanogaster *Tra2 protein, indicating that a fragment of the putative SoTra2 protein had been isolated.

**Figure 1:**
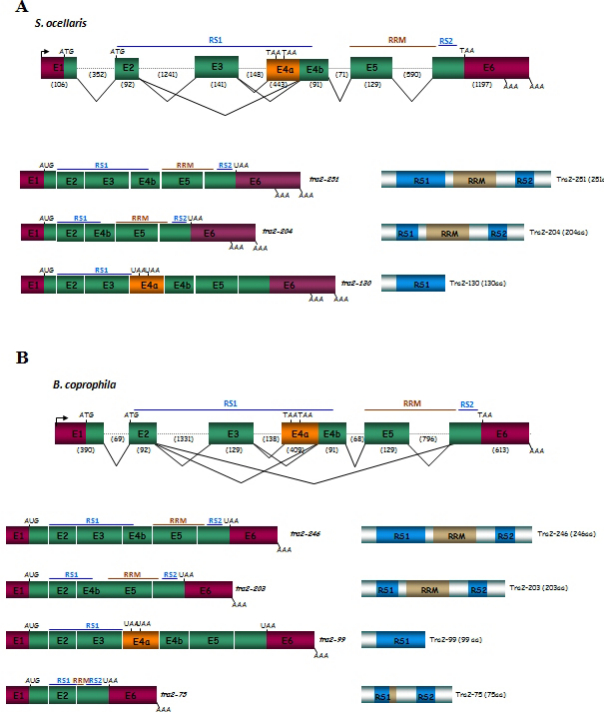
**Molecular organisation of the gene *tra-2 *of *S. ocellaris *(A) and *B. coprophila *(B) and their transcripts**. Exons (boxes) and introns (dotted lines) are not drawn to scale. The colours in the scheme showing the molecular organisation of the gene and in the schemes corresponding to the different mRNA isoforms indicate the 5'- and 3'-UTR regions (in garnet), the protein coding exons (in green) and the specific exon in one of the mRNA isoforms (see text) (in orange). The colours in the scheme showing the proteins designate the RS1 and RS2 domains (in blue) and the RRM domain (in brown). The numbers inside the boxes indicate the number of the exon, while numbers within parenthesis indicate the number of nucleotides composing the exons and introns. The beginning and the end of the ORF are indicated by ATG and TAA respectively. AAA stands for poly-A(+). The arrow indicates the transcription initiation site. RS1, RS2 and RRM denote the corresponding domains of the putative Tra-2 protein.

Nested PCR reactions were then performed in 3' and 5' RACE analyses. The amplicons were then cloned and sequenced. A GenomeWalker library for *S. ocellaris *was then synthesised and used to perform PCR genome-walking on the genomic DNA of *S. ocellaris *from the initial amplicon towards the 5' and 3' directions. The genomic amplicons were cloned and sequenced. The sequences of the genomic fragments thus generated were compared with the cDNA sequences previously determined. In this way, the exon/intron junctions were unambiguously identified. Figure 1A shows the molecular organisation of *Sotra2*. Its transcription unit was made up of 4601 bp and was composed of six exons and five introns (Figure 1A). The transcription start site was located 94 bp upstream of the translation initiation codon.

Overlapping PCR on total RNA of *S. ocellaris *males and females showed that the gene *Sotra2 *mainly produced the transcript *Sotra2-251*, formed by exons E1-E2-E3-E4b-E5-E6 (1177 bp). This encoded a full-length putative Tra2 protein of 251 amino acids that included the RS1, RS2 and RRM domains characteristic of the Tra2 proteins. Two other less abundant transcripts were also detected: *Sotra2-204 *and *Sotra2-130 *(Figure 1A) (see Methods). The *Sotra2-204 *transcript differed from *Sotra2-251 *in its lack of exon 3, and it encoded a putative Tra2 protein of 204 amino acids that differed from the full Tra2 protein in its shorter RS1 domain. The *Sotra2-130 *transcript differed from *Sotra2-251 *by the inclusion of exon E4a. The latter carries translation stop codons causing the production of a truncated Tra2 protein 130 amino acids long and lacking both the RRM and RS2 domains.

To isolate the gene *tra-2 *of *B. coprophila (Bctra2)*, the same strategy used for the isolation of this gene in *S. ocellaris *was followed, except that the initial step involved PCR amplification of the genomic DNA of adults using primers *expSoT2.1 *and *expSoT2.2*, corresponding to exons 5 and 6 of *Sotra2*. A genomic fragment of 1069 bp was amplified, cloned and sequenced. Its sequence showed 61% similarity to the corresponding region of *Sotra2*, suggesting that a genomic fragment of *Bctra2 *had been cloned. As for *Sotra2*, 5'RACE, 3'RACE and genome-walking methodologies were used to determine the molecular organisation of *Bctra2 *(Figure 1B). Its transcription unit was made up of 4255 bp and was composed of six exons and five introns.

Overlapping PCR performed on total RNA of males and females of *B. coprophila *showed that the gene *Bctra2 *mainly produced the transcript *Bctra2-246*, formed by exons E1-E2-E3-E4b-E5-E6 (1444 bp). This encoded a full putative Tra2 protein of 246 amino acids and included the RS1, RS2 and RRM domains characteristic of Tra2 proteins. Three other less abundant transcripts were also detected: *Bctra2-203*, *Bctra2-99 *and *Bctra2-75 *(Figure 1B) (see Methods). The *Bctra2-203 *transcript differed from *Bctra2-246 *in its lack of exon 3; it encoded a putative Tra2 protein of 203 amino acids. The *Bctra2-99 *transcript varied from *Bctra2-246 *by the inclusion of exon E4a, which carries translation stop codons. Thus, it produced a truncated Tra2 protein of 99 amino acids that lacked both the RRM and RS2 domains. Finally, the *Bctra2-75 *transcript, made up by exons E1, E2 and E6, encoded a truncated Tra2 protein with short RS1, RRM and RS2 domains.

### Expression pattern of *tra-2 *in *S. ocellaris *and *B. coprophila*

**Figure 2:**
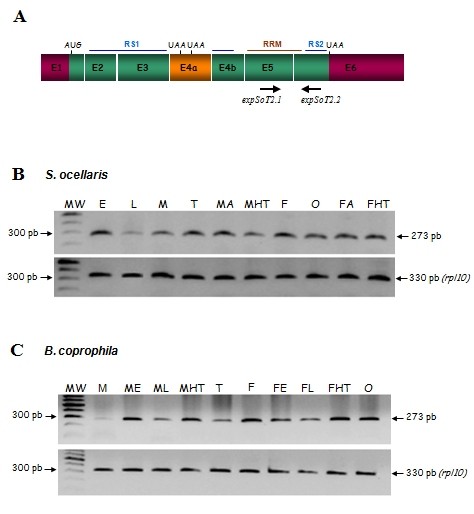
**Expression of *tra-2 *of *S. ocellaris *and *B. coprophila***. **(A) **Molecular organisation (only exons are shown) of the *Sciara tra-2 *transcript with the primers used. The meaning of the colours is explained in the legend to Figure 1. **(B) **RT-PCR analyses of total RNA from *S. ocellaris *embryos (E), larvae (L), whole male adults (M), testis (T), adult male abdomens (MA), adult male head plus thorax (MHT), whole adult females (F), ovaries (O), adult female abdomens (MA), and adult female head plus thorax (MHT). **(C) **RT-PCR analyses of total RNA from *B. coprophila *whole male adults (M), male embryos (ME), male larvae (ML), adult male head plus thorax (MHT), testis (T), whole female adults (F), female embryos (FE), female larvae (FL), adult female head plus thorax (FHT) and ovaries (O). Protein L10 was used as a control of the expression of the gene *rpL10*, which codes for the constitutive ribosomal protein.

The expression of *tra2 *in *S. ocellaris *was studied by performing RT-PCR on total RNA from a mixture of male plus female embryos, from a mixture of male plus female larvae at different developmental stages, from the heads plus thoraces of male and female adults (separately), from the abdomens of male and female adults (separately), and from adult ovaries and testis (separately). The expression of *tra2 *in *B. coprophila *was similarly analysed, although in this case it was possible to distinguish male from female embryos as well as male from female larvae (see Methods). The primers used were *expSoT2.1 *from exon 5 and *expSoT2.2 *from exon 6 (Figure 2A), which have the same sequence in both *Sciara *species. The expression of the constitutive gene *rpL10*, which encodes the ribosomal protein L10, was used as a control in RT-PCR. In all cases, a fragment of 273 bp was amplified (Figure 2B,C). This was cloned and sequenced, confirming that it corresponded to the expected *Sotra2 *or *Bctra2 *fragment. Negative controls in all these PCR reactions produced no amplicons (see Methods). These results indicate that the genes *Sotra2 *and *Bctra2 *are expressed at all developmental stages and during adult life in both sexes, including in the gonads of males and females.

### Comparison of the molecular organisation of *Sciara tra-2 *gene with that of other insects

The gene *tra-2 *of *D. melanogaster *gives rise to three mRNAs by alternative splicing pathways and alternative promoters, which encode three distinct isoforms of the Tra2 protein [27,28]. In *B. mori*, *tra-2 *produces six different transcripts by alternative splicing pathways, which encode six distinct isoforms of the Tra2 protein [24]. In the case of other dipterans such as *C. capitata *[20,21], *Anastrepha *species [22], *M. domestica *[19] and *L. cuprina *[23], only a single *tra-2 *mRNA was detected.

**Figure 3:**
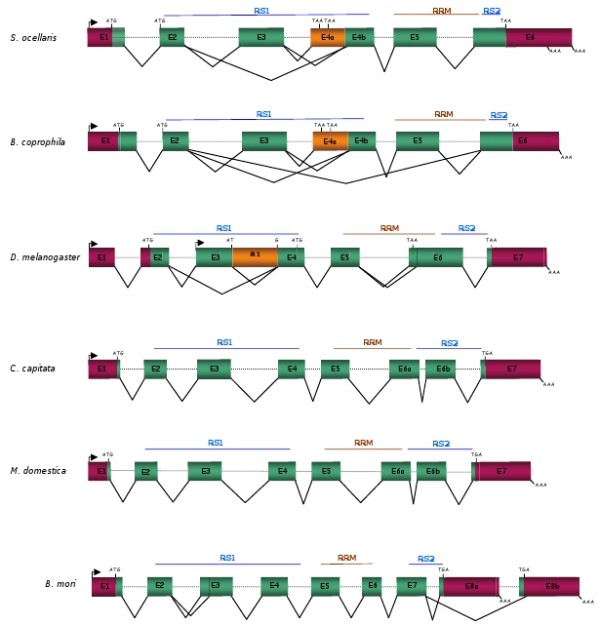
**Comparison of the molecular organisation of *tra-2 *of *S. ocellaris, B. coprophila, D. melanogaster, C. capitata, M. domestica *and *B. mori***. Exons (boxes) and introns (dashed lines) are not drawn to scale. For the rest of symbols and colours see legend to Figure 1.

Figure 3 compares the molecular organisation of *Sciara tra-2 *with *tra-2 *of *Drosophila, Ceratitis, Musca *and *Bombyx*. The number of exons varies: six in *Sciara*, seven in *Drosophila*, eight in *Ceratitis *and *Musca*, and nine in *Bombyx*. In *Sciara*, all the exon/intron junctions agree with the consensus GT/AG. These joints are found in the same position in *S. ocellaris *and *B. coprophila*. Similarly, the connection between exons 5 and 6 is in the same position in all these species. The RS1 and RRM domains of the putative Tra2 proteins are encoded by the exons E2-E4 and exons E5-E6 respectively in all species. However, the RS2 domain is encoded by exon E6 in *Sciara*, by exons E6-E7 in *Drosophila*, by exons E6b-E7 in *Ceratitis*, by exons E6-E7 in *Musca*, and by exons E7-E8 in *Bombyx*. The gene *tra-2 *has a single promoter except in *Drosophila*, which carries two promoters.

### Comparison of the Tra2 protein of *Sciara *with that of other insects

The conceptual translation of the *Sotra2-251 *and *Bctra2-246 *mRNAs showed them to encode a polypeptide with the main structural features characteristic of the SR protein family, i.e., the RNA-binding motif (RRM) and two RS-domains. These are rich in serine-arginine dipeptides and confer upon these proteins the capacity to interact with others.

**Figure 4:**
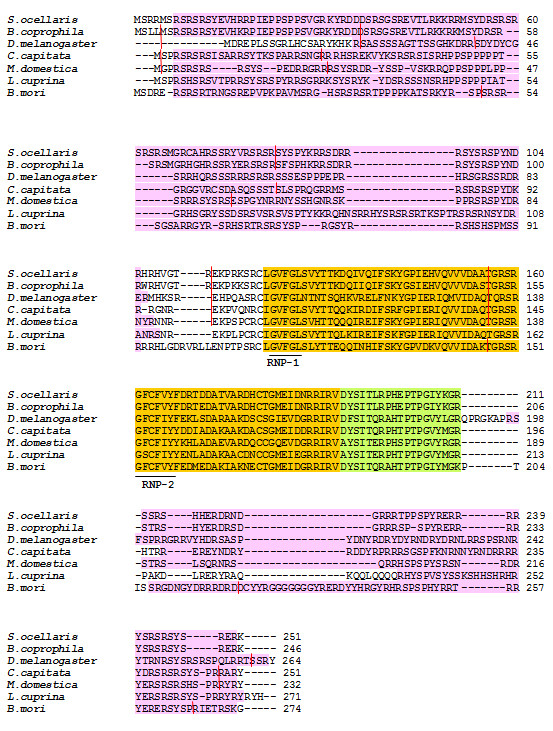
**Comparison of the Tra-2 predicted polypeptides of the *Sciara *species with Tra-2 of other insects**. The RS1 and RS2 domains are in pink, the RRM domain is in yellow and the linker region is in green. RNP1 and RNP2 are the consensus sequences for RNA binding. The red vertical lines indicate the exon-intron junctions. Accession numbers: *S. ocellaris *[EMB: FR716529], *B. coprophila *[EMB: FR716530], *D. melanogaster *[EMBL:M23633], *C. capitata *[EMBL:EU999754], *M. domestica *[EMBL:AY847518], *L. cuprinia *[EMBL:FJ461620] and *B. mori *[EMBL:NM_001126233]

The putative Tra2 protein of the dipterans *S. ocellaris *and *B. coprophila *(Sciaridae, suborder Nematocera) were compared to those of the dipterans belonging to the suborder Brachycera, i.e., *D. melanogaster *(Drosophilidae)*, C. capitata *(Tephritidae), *M. domestica *(Muscidae) and *L. cuprina *(Calliphoridae), and that of the lepidopteran *B. mori*. Figure 4 shows their alignment. The number of amino acids in these Tra2 proteins varied: *S. ocellaris *251, *B. coprophila *246, *D. melanogaster *264, *C. capitata *251, *M. domestica *232, *L. cuprina *271, and *B. mori *274. These differences are due to changes throughout the Tra2 protein except in the RRM domain and the linker region (72 and 19 amino acids respectively) in all these species. The highest degree of similarity (measured as identical plus conserved amino acids) to *S. ocellaris *was shown by *B. coprophila *(86%) (as expected for species belonging to the same Family), followed by *Musca *(46.9%), *Bombyx *(44.6%), *Ceratitis *(42.6%), *Lucilia *(39,4%) and *Drosophila *(36.3%). The highest degree of similarity was observed in the RRM (55.5-94.4%) and the linker region (68.4-100%). In fact, the linker region is a signature motif of Tra2 proteins [29]. This conservation agrees with the fundamental role of RRM in the function of the Tra-Tra2 complex, conferring upon the complex its capacity to interact with the *tra *and *dsx *pre-mRNAs and thus regulate its sex-specific splicing. The similarity of the RS1 (21.8-87.6%) and RS2 (28.2-81.6%) domains was lower and a variable number of SR dipeptides were seen, ranging from 11 to 18 for RS1 and 3 to 6 for RS2. Variation in the content of RS dipeptides seems to be a feature of the SR proteins whenever they are maintained enough to preserve their function [30].

### Effect of the gene *tra-2 *of *Sciara *on the somatic sexual development of *Drosophila*

Outside *Drosophila*, the function of *tra-2 *in sex determination has been unambiguously demonstrated in *M. domestica *[19], in *C. capitata *[21] and in *Anastrepha *[22] using the interference-RNA technique, which permits functional studies of genes in genetically less amenable organisms. An imperative of this technique is to have markers that allow one to determine whether male survivors really do correspond to XX females that have been transformed into pseudomales by the destruction of the endogenous *tra-2 *gene function, or to normal XY males. In the case of the insects mentioned above, this distinction was possible thanks to the existence of molecular markers located on the Y chromosome [19,21] and to the different morphology of the X and Y chromosomes [22]. However, the lack of molecular makers, plus the fact that *Sciara *females are XX and males are X0, together with the extreme fragility of their tiny eggs, makes this RNAi procedure unfeasible for these insects at the present time. Thus, direct proof of the role of *tra-2 *in *Sciara *sex determination remains elusive.

Notwithstanding, we were able to study whether the *Sciara *Tra2 protein shows conserved sex-determination function in *Drosophila*. The rationale of the experiment was to express transgenic SoTra2 protein in *Drosophila *XX pseudomales lacking the *tra-2 *gene function and checked whether these pseudomales showed feminisation. The GAL4-UAS system was used to analyse the effect of the *Sciara tra-2 *gene in *Drosophila*.

The systemic expression of SoTra2 with the ubiquitous-expression *da-GAL4 *or *hs-GAL4 *drivers was found to be lethal to both male and female flies. The same lethality has been observed in *Drosophila *males and females that ectopically express their own Tra2 protein [31]. Therefore, the *rn-GAL4 *local expression driver was used. This driver expresses GAL4 in agreement with the expression domain of the gene *rotund (rn)*, which is expressed in the tarsal region of the foreleg imaginal disc [32], a well-characterised sexually dimorphic region of *Drosophila*. For details of the experimental design see Methods.

**Figure 5:**
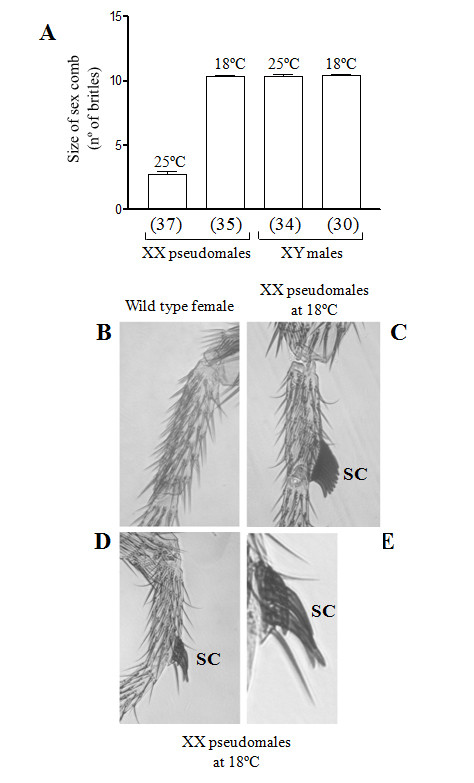
**Effect of the *Sciara tra-2 *gene on the somatic development of *Drosophila***. **(A) **Size (number of bristles) of the sex comb in the *Sotra2 *transgenic *Drosophila *flies. The bars in the histograms represent the 95% confidence limits. The sample size is given in parenthesis underneath the histogram. The full genotype of XX pseudomales is *ywTDSo#7/w; tra-2^B^/Df(2R)trix,tra-2*^-^*; Tub-GAL80^ts^,w^+^/rn-GAL4,w^+ ^*and that of their brothers is XY males *ywTDSo#7/Y; tra-2^B^/Df(2R)trix,tra-2*^-^*; Tub-GAL80^ts^,w^+^/rn-GAL4,w^+^*. The cross to generate these flies was: females *ywTDSo#7; tra-2^B^/CyO,Cy; Tub-GAL80^ts^,w^+ ^*and males *w/Y; Df(2R)trix,tra-2*^-^*/CyO,Cy; rn-GAL4,w^+^*. **(B) **Foreleg basitarsus of wild type female. **(C) **Foreleg basitarsus of XX pseudomale mutant for *tra-2 *at 18°C, in which the *Sciara *Tra-2 is not expressed. **(D) **Foreleg basitarsus of XX pseudomale mutant for *tra-2 *at 25°C, in which the *Sciara *Tra-2 is partially expressed. **(E) **Enlargement of D. SC stands for sex comb.

Figure 5 shows the effect of expressing the SoTra2 protein on the sexually dimorphic development of the foreleg basitarsus in XX pseudomales and in their brother XY males, both mutant for *tra-2 *and carrying *Sotra2-UAS*, *rn-GAL4 *and *Tub-GAL80^ts^*. The foreleg basitarsus contained several transversal rows, the last one forming the sex comb structure (SC) in males and in XX pseudomales mutants for *tra-2*. This is composed of dark, thick bristles, and is rotated to lie parallel to the proximal-distal leg axis (Figure 5C). Females lack the sex comb (Figure 5B). A significant reduction (P < 0.0001, one-way ANOVA) was seen in the number of bristles forming the male sex comb structure in the foreleg basitarsus of XX pseudomales raised at 25°C (expressing the SoTra2 protein) (see Figure 5D and the enlargement in Figure 5E) compared to those raised at 18°C (no expression of the SoTra2 protein) (see Figure 5C). The sex comb size of these latter pseudomales was the same as the sex comb of their XY brothers whether raised at 18 or 25°C (Figure 5A). Thus, the *Sciara *Tra-2 protein supplies *tra-2 *function in *Drosophila*.

This reversion of the male towards the female phenotype of the foreleg basitarsus in XX pseudomales mutant for *tra-2 *and expressing the SoTra2 protein is probably caused by the presence of the endogenous *Drosophila *DsxF protein. As mentioned in the Introduction, the Tra-Tra2 complex controls the female-specific splicing of the *dsx *pre-mRNA. If the *Sciara *Tra2 protein is capable of forming a complex with the endogenous *Drosophila *Tra protein, then this complex could bind to specific sequences in the female-specific exon of *dsx *pre-mRNA. This would promote its inclusion in mature *dsxF *mRNA, which encodes the DsxF protein that establishes female development. This expectation was confirmed at the molecular level.

**Figure 6:**
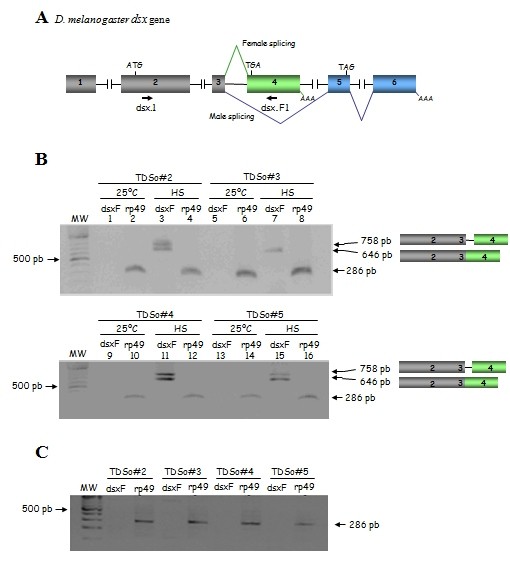
**Effect of the *Sciara tra-2 *gene on the splicing of *Drosophila dsx *pre-mRNA**. **(A) **Molecular organisation of *Drosophila dsx *pre-mRNA showing the male and the female splicing pattern. Exons are represented in boxes and introns by dotted lines. The female-specific exon is showing in green and the male-specific exons are shown in blue. **(B) **RT-PCR analyses of total RNA from *D. melanogaster *XX pseudomales mutant for *tra-2 *and either not expressing (25°C) or expressing (HS) the *Sciara tra-2 *transgene. *TDSo#2 *stands for XX pseudomale of genotype *yw/w; tra-2^B^/Df(2R)trix,tra-2^-^; TDSo#2/hs-GAL4,w^+^*; *TDSo#3 *stands for XX pseudomale of genotype *yw/w; tra-2^B^/Df(2R)trix,tra-2^-^; TDSo#3/hs-GAL4,w^+^; TDSo#4 *stands for XX pseudomale of genotype *yw/w; tra-2^B^/Df(2R)trix,tra-2^-^; TDSo#4/hs-GAL4,w^+ ^*and *TDSo#5 *stands for XX pseudomale of genotype *yw/w; tra-2^B^/Df(2R)trix,tra-2^-^; TDSo#5/hs-GAL4,w^+^*. **(C) **RT-PCR analyses of total RNA from *D. melanogaster *XY males mutant for *tra-2 *(brothers of the XX pseudomales) expressing the *Sciara tra-2 *transgene. *TDSo#2 *stands for XY male of genotype *yw/Y; tra-2^B^/Df(2R)trix,tra-2^-^; TDSo#2/hs-GAL4,w^+^*; *TDSo#3 *stands for XY male of genotype *yw/Y; tra-2^B^/Df(2R)trix,tra-2^-^; TDSo#3/hs-GAL4,w^+^; TDSo#4 *stands for XY male of genotype *yw/Y; tra-2^B^/Df(2R)trix,tra-2^-^; TDSo#4/hs-GAL4,w^+ ^*and *TDSo#5 *stands for XY male of genotype *yw/Y; tra-2^B^/Df(2R)trix,tra-2^-^; TDSo#5/hs-GAL4,w^+^*. The cross to generate these XX pseudomales and XY males flies was: females *yw; tra-2^B^/CyO,Cy; TDSo# *and males *w/Y; Df(2R)trix,tra-2^-^/CyO,Cy; hs-GAL4,w^+^*. As a control, the splicing of the primary transcript of gene *rp49 *that codes for the constitutive ribosomal protein 49 [33] was monitored.

The effect of *Sciara *Tra2 protein on the splicing control of *Drosophila dsx *pre-mRNA was studied in transgenic *Drosophila *XX flies mutant for *tra-2 *and expressing the *Sciara *Tra2 protein (Figure 6). The inducible *hs-GAL4 *driver was used to express the *Sotra2 *transgene. XX pseudomales *yw/w; Df(2R)trix,tra-2[-]/tra-2^B^; Sotra2/hs-GAL4 *were produced at 25°C (see cross in the legend to Figure 6). After the hatching of the adults the flies were divided into two populations; one was maintained at 25°C (control flies) and one subjected to heat-shock pulses (experimental flies) to induce the expression of the *Sotra2 *transgene. Total RNA was extracted and used in RT-PCR to determine the splicing pattern of the endogenous *dsx *primary transcript. *rp49*, which codes for the constitutive ribosomal protein 49 [33], was used as an RT-PCR control. At 25°C, the four transgenic lines only expressed the male *dsx *mRNA isoform (data not shown); this was to be expected since they lack the endogenous *tra-2 *function and do not express the *Sotra2 *transgene. After the heat shocks, however, these transgenic lines expressed the female *dsx *mRNA isoform (Figure 6B). Two amplicons were detected. The smaller one (646 bp) corresponded to the female *dsxF *mRNA. The larger amplicon (758 bp) was to be expected if the intron 3 were retained (Figure 6A,B). The cloning and sequencing of both fragments confirmed these suppositions. These results were not the consequence of the heat-shocks since their brothers (males *yw/Y; Df(2R)trix,tra2[-]/tra-2^B^; Sotra2/hs-GAL4*) expressing the *Sotra2 *transgene did not express the female *dsx *mRNA isoform (Figure 6C). Negative controls for all these PCR reactions produced no amplicons (see Methods). Thus, the *Sciara *Tra2 protein is able to promote the female-specific splicing of the *Drosophila dsx *pre-mRNA.

Whereas the expression of *Sciara *Tra2 protein in the XX pseudomales produced their feminisation, its expression in their XY normal brothers mutant for *tra-2 *did not affect their normal male development. This different effect is explained by the presence of Tra protein in the XX pseudomales and its absence in XY normal males. The *Drosophila *Tra protein seems to lack an RNA binding domain, thus its influence in female development is exerted at the level of its interaction (via SR domains) with other proteins carrying RNA-binding domains, such as Tra2 (reviewed in [34]). Therefore, the *Sciara *Tra2 protein would form a complex with the endogenous *Drosophila *Tra protein to promote the female-specific splicing of the *Drosophila dsx *pre-mRNA.

The feminisation produced by the *Sciara *Tra2 protein was, however, partial, indicating that the function of this protein in *Drosophila *was incomplete. There are two possible explanations for this. It might be due to an insufficient quantity of *Sciara *Tra2 protein being produced in the *Drosophila *transgenic flies; it was necessary to restrict the amount of *Sciara *Tra2 protein that was expressed since the production of any greater amount is lethal. However, although this possibility cannot be rejected outright it seems unlikely since *Drosophila *XX flies with a single dose of *tra-2 *develop as normal females; i.e., a single dose of the endogenous *tra-2 *gene supplies enough *Drosophila *Tra2 protein for normal female development to be followed. Alternatively, the interaction between the endogenous *Drosophila *Tra protein and the transgenic *Sciara *Tra2 protein might be affected such that the *Drosophila*Tra-*Sciara*Tra2 complex is less efficient than the *Drosophila*Tra-Tra2 complex at inducing the female-specific splicing of the endogenous *Drosophila dsx *pre-mRNA. This explanation agrees with the presence of the aberrant spliced *dsxF *mRNA isoform in XX pseudomales expressing the *Sciara *Tra2 protein in addition to the normally spliced *dsxF *mRNA. Note that this isoform contains intron 3, the target where the Tra-Tra2 complex binds to promote its inclusion into mature *dsxF *mRNA [7-10]. Further, the retention of intron 3 does not appear to be the consequence of any general trouble in the splicing of *dsx *pre-mRNA since intron 2 (and probably also intron 1) is normally spliced.

This aberrant *dsxF *mRNA isoform has been also found in *Drosophila *XX pseudomales mutant for the endogenous *tra *gene and expressing the *Anastrepha *Tra protein, whereas *Drosophila *XX flies with different doses of the endogenous *tra *and *tra-2 *genes do not show this abnormally spliced *dsxF *mRNA isoform [35]. With respect to the proposed inefficient interaction between the endogenous *Drosophila *Tra protein and the transgenic *Sciara *Tra2 protein, the high degree of divergence between the *Sciara *and the *Drosophila *Tra2 proteins should be noted. This divergence was mainly observed in the RS domains, which are involved in protein-protein interactions. Hence, the interaction between the *Sciara *Tra2 protein and the *Drosophila *Tra protein might be impeded as a consequence of changes accumulated in these proteins after the *Sciara *and *Drosophila *phylogenetic lineages separated. These results suggest that Tra and Tra2 proteins co-evolved to exert their functions in sex determination. To this respect, it is worth mentioning that the *D. virilis tra-2 *gene can fully replace the endogenous *tra-2 *function of *D. melanogaster *for normal female sexual development [18]. The similarity between the *D. melanogaster *and *D. virilis *Tra-2 proteins is 51,5% [22], whereas the similarity between the *D. melanogaster *and *S. ocellaris *Tra-2 proteins is 36.3% [this work].

## Conclusions

The *transformer-2 *genes of both *Sciaridae *species encode a single protein in both sexes that shares the characteristics of the Transformer-2 proteins of other insects. These proteins showed conserved sex-determination function in *Drosophila*; i.e., they were able to form a complex with the endogenous *Drosophila *Transformer protein that controls the female-specific splicing of the *Drosophila doublesex *pre-mRNA. However, it appears that the complex formed between the *Drosophila *Transformer protein and the *Sciara *Transformer-2 protein is less effective at inducing the female-specific splicing of the endogenous *Drosophila doublesex *pre-mRNA than the *Drosophila*Transformer-Transformer2 complex. This suggests the existence of species-specific co-evolution of the Transformer and Transformer-2 proteins.

## Methods

### Flies and crosses

*Sciarid *flies were raised on "Compost Villacasa" medium for culturing mushrooms. *S. ocellaris *is a digenic species so that females produce both sexes, whereas *B. coprophila *is a monogenic species with two types of females: gynogenic females, which produce only females, and androgenic females, which produce only males.

*Drosophila *flies were cultured on standard food. For the description of the mutant alleles and GAL4 constructs see Lindsley and Zimm [36] and FlyBase. Flies used for the analysis of the adult forelegs were kept in a mixture of ethanol:glycerol (3:1) for several days. They were then macerated in 10% KOH at 60°C for 15 min, thoroughly washed with water, and mounted in Faure's solution for inspection under a compound microscope.

### Experimental design to analyse the effect of the gene *tra-2 *of *Sciara *on the somatic sexual development of *Drosophila*

As mentioned in the text, the systemic expression of SoTra2 with the ubiquitous-expression *da-GAL4 *or *hs-GAL4 *drivers was found to be lethal to both male and female flies. Therefore, the *rn-GAL4 *local expression driver was used. Nevertheless, it was necessary to find out the experimental conditions that allowed us to study the effect of the gene *tra-2 *of *Sciara *on the somatic sexual development of *Drosophila*. The *rn-GAL4 *driver expresses GAL4 in agreement with the expression domain of the gene *rotund (rn) *[32]. This gene is expressed in imaginal discs as well as in the embryonic and larval central nervous systems (CNS) [32]. The expression of *rn *in the tarsal region of the foreleg imaginal disc commences during the early third larval instar, but is no longer evident in the late third instar [32]. This methodology has been used to ablate the sex comb in *Drosophila *males expressing the Transformer protein under the *rn-GAL4 *driver [37]. The expression of *Sotra2 *by the *rn-GAL4 *driver was also lethal to both males and females. This might be due to the expression of *Sotra2 *in the embryonic and/or larval CNS, as mentioned above. To prevent embryonic lethality a strategy was followed that allowed the temporal control of *Sotra2 *expression under the *rn-GAL4 *driver during development. For this purpose the GAL4/GAL80 system was used. The GAL80 protein inhibits GAL4 function. GAL80 is temperature sensitive, with 18°C the most permissive temperature and 29°C the most restrictive [38]. *Drosophila *XX pseudomales mutant for *tra-2 *and carrying *Sotra-2-UAS *together with *rn-GAL4 *and *Tub-GAL80^ts ^*were produced. The cross producing these pseudomales was performed at 18°C, and several two-days egg collections were made. Three days later each collection was transferred to 29°C environment. By this time the larvae had hatched, which were maintained at this temperature for the rest of their development. This treatment eliminated the embryonic lethality caused by the expression of the SoTra2 protein; at 18°C the *UAS-Sotra2 *transgen is not expressed since the GAL4 protein from the *rn-GAL4 *driver is not functional due to the function of GAL80^ts ^protein. However, the expression of *Sotra2 *during the larval stage could not be prevented since at 29°C this transgene is expressed due to the inactivation of the GAL80^ts ^protein. Even under these conditions, the males and females expressing the SoTra2 protein were lethally affected, probably because of an excess of this transgenic protein affecting the development of the larval CNS. Finally, we took advantage of the fact that at 25°C the GAL80^ts ^protein retains some function [38]. It was reasoned that if XX pseudomales mutant for *tra-2 *and carrying *Sotra-2-UAS*, *rn-GAL4 *and *Tub-GAL80^ts ^*can develop at that temperature, the GAL4 from the *rn-GAL4 *driver ought not to be completely inhibited. Consequently a certain amount of SoTra2 protein ought to be produced (less than at 29°C) but which might not be lethal. Thus, these pseudomales might be able to survive to adulthood. This was the case.

### Molecular analyses

Genomic DNA was extracted from frozen specimens as described in Maniatis et al. [39]. Total RNA from adult female ovaries, adult male testis, embryos, larvae, and adult male and female somatic cells was prepared using the Ultraspec-II RNA isolation kit (Biotecx) following the manufacturer's instructions. Five micrograms of total RNA from each sample were reverse transcribed with Superscript (Invitrogen) following the manufacturer's instructions. Reverse transcription reactions were performed with an oligo-dT. Two percent of the synthesised cDNA was amplified by PCR. All amplicons were analysed by electrophoresis in agarose gels, cloned using the TOPO TA-cloning kit (Invitrogen) or PGEM-T vector (Promega) following the manufacturer's instructions, and sequenced. In all cases, PCR reactions with RNA samples were performed to guarantee there was no contamination with genomic DNA (negative controls of PCR reactions). The relative abundance of distinct mRNA isoforms was inferred from the different intensity of their corresponding amplicons in the agarose gels showing the products of the RT-PCR reactions. In some cases, the amplicons were not clearly visible in the gel.

The GenomeWalker genomic libraries of *S. ocellaris *and *B. coprophila *were synthesised using the BD GenomeWalker Universal kit (BD Biosciences), following the manufacturer's instructions.

For the construction of the *Sotra2 *transgene, the *tra-2 *ORF of *S. ocellaris *was amplified by RT-PCR. The PCR reaction was performed using primers *5'SoT2 *and *3'SoT2*. The resulting amplicon was cloned in *pUAST*. The microinjections for generating the *TDSo *(*UAS::Sotra-2 *cDNA) transgenic *D. melanogaster *lines were performed by Genetic Services (Sudbury, MA, USA). To ascertain that each transgenic line was carrying the correct transgene, PCR on genomic DNA was used to amplify the whole transgene. The amplicons were then cloned and sequenced.

### DNA sequencing

Sequencing was performed using an automated 377 DNA sequencer (Applied Biosystems). The analysis of DNA and protein sequences was performed using the programmes DNA Compare, Editview and BioEdit, and information in NCBI http://www.ncbi.nlm.nih.gov, BLAST http://www.ncbi.nlm.nih.gov/BLAST, FlyBase http://flybase.org/, CLUSTALW http://www.ebi.ac.uk/clustalw and FASTA http://www.ebi.ac.uk/Tools/fasta33/index.htm databases.

## Author's contributions

IK performed the experiments. MFR supervised the molecular biology experiments. LS conceived and supervised the study, and wrote the manuscript. All authors contributed to the final version of the manuscript.

## Supplementary Material

Additional File 1 **Location and sequences of the primers used in this work**. The arrows indicate the location of the primers in the schemes showing the molecular organisation of the genes *tra-2 *of *S. ocellaris *and *B. coprophila*. The meaning of the colours is explained in the legend to Figure 1 Click here for file
