# Supplementary figures and images for: The gene transformer-2 of Sciara (Diptera, Nematocera) and its effect on Drosophila sexual development
Source: BMC Dev Biol. 2011 Mar 15;11:19. doi: 10.1186/1471-213X-11-19 (PMC3068122; doi:10.1186/1471-213X-11-19)

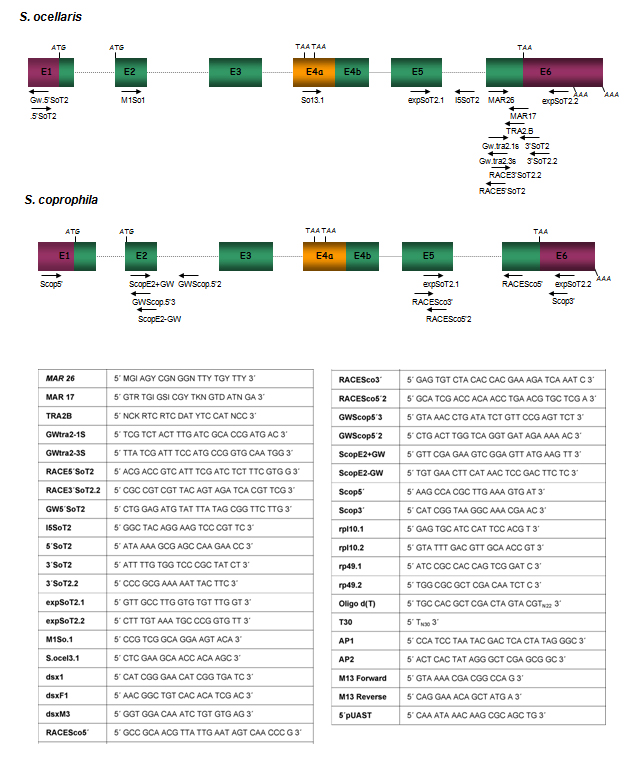

Supplement: Additional File 1 — Location and sequences of the primers used in this work. The arrows indicate the location of the primers in the schemes showing the molecular organisation of the genes tra-2 of S. ocellaris and B. coprophila. The meaning of the colours is explained in the legend to Figure 1 [file 1471-213X-11-19-S1.JPEG]
